# Measurements of Liquid Ammonia Sprays Using the ECN Spray M Injector with Injector-To-Injector Comparisons

**DOI:** 10.1021/acs.energyfuels.6c00572

**Published:** 2026-07-12

**Authors:** Li Shen, Abdullah U. Bajwa, Felix Leach

**Affiliations:** † Department of Engineering Science, 6396 University of Oxford, Parks Road, Oxford OX1 3PJ, U.K.; ‡ 7072 Southwest Research Institute, 9503 W Commerce, San Antonio, Texas 78227-1301, United States

## Abstract

Liquid ammonia is receiving increasing attention as a carbon-free energy carrier for use in internal combustion engines, particularly in the maritime sector. While gaseous ammonia injection has been widely studied, liquid-phase injection offers potential advantages in terms of fuel–air mixing and power density, but introduces challenges associated with evaporation, flash boiling, and spray collapse. This study presents a comprehensive experimental investigation of liquid ammonia sprays generated using a multihole ECN Spray M injector and a commercially available injector. Experiments were conducted in a nitrogen-filled constant volume chamber over a wide range of ambient pressures (1–15 bar) and at two injection pressures (100 and 150 bar). High-speed shadowgraph imaging was used to characterize spray morphology and macroscopic spray parameters, including tip penetration length, tip penetration speed, and spray cone angle, following the SAE J2715 standard. In parallel, instantaneous mass flow rate was measured using a pressure-based technique upstream of the injector, enabling quantification of injected mass under the tested conditions. The results show that ammonia spray behavior is strongly governed by the saturation-to-ambient pressure ratio (*r*_p_), with three distinct regimes identified: drag-dominated, evaporation-dominated, and flare flash boiling. Compared to the commercial injector, the ECN Spray M injector exhibits an earlier onset of spray collapse at lower *r*_p_ values, attributed to differences in injector geometry, particularly the longer counterbore promoting in-nozzle cavitation. Despite these morphological differences, the mass flow rate and total injected mass from the ECN Spray M injector were found to be largely insensitive to ambient pressure, in contrast to previous observations with other injector designs. This work presents the first open-literature data set of mass flow rate and high-resolution macroscopic spray parameters for the multihole ECN Spray M injector under flashing conditions. The results provide valuable insights into injector design effects on ammonia fuel injection and offer a robust experimental benchmark for future computational model validation.

## Graphical abstract

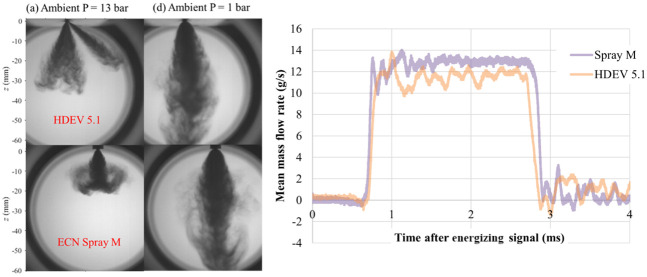

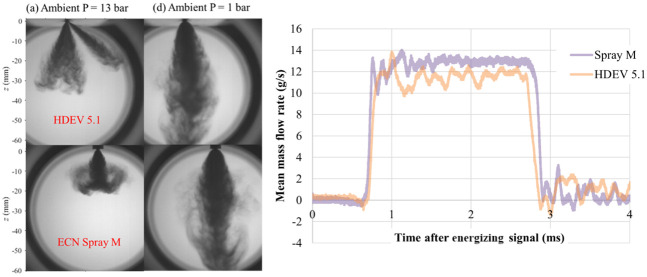

## Introduction

Maritime transport contributes over one billion tonnes of CO_2_ emissions annually, accounting for approximately 2.9% of global emissions.^1^ As a carbon-free energy carrier, ammonia has gained considerable attention as a promising alternative fuel for decarbonizing the maritime sector.^2^ Ammonia is under consideration for use in internal combustion engines, primarily for the maritime (shipping) sector and the energy storage (stationary power generation) sector.^3^

When used in an internal combustion engine, ammonia can either be injected in the gas phase or the liquid phase, and so far, most applications have focused on gaseous ammonia.^4–6^ However, liquid-phase ammonia injection offers distinct advantages, including enhanced fuel-air mixing due to the high momentum of liquid droplets and the potential for achieving higher power densities.^7^ However, the high enthalpy of vaporization of ammonia poses significant challenges for evaporation as liquid ammonia is injected, often resulting in the presence of liquid droplets during combustion, which can negatively affect combustion efficiency and emissions. Recent experimental studies have begun to explore the spray characteristics of liquid ammonia. Pele et al. demonstrated that ammonia sprays, generated using a repurposed gasoline direct injection (GDI) system, exhibit longer penetration lengths and narrower spray cone angles than gasoline sprays, with strong sensitivity to ambient conditions.^8^ Similarly, Cheng et al. reported that ammonia sprays mix more rapidly with air and have broader spray cone angles compared to methanol and ethanol.^9^ Additional investigations by Colson et al. identified a transition regime toward flash boiling,^10^ while Shen et al. highlighted the significant influence of ambient pressure on ammonia spray morphology.^11^ Despite these insights, spray collapse, a phenomenon common in other fuels at reduced ambient pressures, is only rarely observed in ammonia sprays, even at ambient-to-saturation pressure ratios as low as 0.35.^12–14^ A number of injector design parameters are known to influence flash boiling, such as divergent nozzle holes resisting spray collapse,^15^ long nozzles, and sharp nozzle inlets promoting bubble (and hence flash boiling) formation.^16^ Theoretically, flash boiling (the source of spray collapse) would occur at ambient-to-saturation pressure ratios below 1. Recently, Shen et al.^17^ showed that the macroscopic behavior of ammonia sprays can be divided into three regions, namely, the drag-dominant, evaporation-dominant, and spray collapse or flare flash boiling regions.

The Engine Combustion Network (ECN) is a long-standing international collaborative research effort that provides experimental data and a framework to improve Computational Fluid Dynamics (CFD) models for engine combustion. By using the same injectors, which are well-characterized and documented, and the same experimental conditions, a large library of experimental data appropriate for model validation is achieved.^18^ The ECN has several different injectors that it uses, but a main focus for ammonia sprays has been the ECN Spray M injector. The ECN Spray M injector is very closely based on a previous GDI injector design (ECN Spray G). It exists in two versions, a single-hole injector, where data has been reported in Bjørgen et al., Desclaux et al., and Sonawane et al.^19–21^ and a multihole version, which is used in this work, where data has been reported in a single study, Qenawy et al.^22^ Qenawy et al.^22^ tested the injector at only two flash boiling conditions—their study focusing mainly on supercritical conditions. They did find that spray collapse occurred in low-pressure high-temperature conditions close to the critical point.

Mass flow rate is a key input parameter to all CFD simulations of sprays.^23–25^ Without accurate knowledge of the mass flow rate for CFD simulations, it becomes just another tuning parameter that needs to be adjusted to match parameters of interest (such as spray liquid penetration length). Hence, accurate mass flow rate data for ammonia sprays are a key piece of information that needs to be made available as research on ammonia as an energy vector increases. Because ammonia has a relatively high saturation pressure, when injected, it almost immediately exists in two phases (gas and liquid), which makes traditional mass flow measurement techniques, such as the Bosch or Zeuch methods, unusable. However, recently, a technique based on momentum flux by Bracho and coworkers,^26^ has enabled mass flow rate measurements on ammonia injections for the first time. This work presents mass flow rate measurements using an alternative: a pressure-based method, based on the work of Ferrari et al.^27^ adapted for use with ammonia.

Ferrari and colleagues^27^ proposed a diesel injection control strategy based on pressure measurements within the injector pipeline that links the fuel rail and injector. By measuring this pressure, the mass flow rate could be determined, enabling correction of the injected fuel mass in a diesel engine. The approach employs the pressure-time profile within the pipe to determine the instantaneous fuel mass flow rate using either one or two pressure measurement locations. For small injection quantities, a single pressure measurement location was sufficient, whereas larger injections required measurements at both locations to account for pressure wave reflections arising during extended injector opening durations.

In this work, ammonia sprays from a multihole ECN Spray M injector are evaluated. Mass flow rate measurements using a pressure-based method are presented alongside macroscopic spray parameters such as spray tip penetration length, tip penetration speed, and spray cone angle are reported. This is the first report in the open literature of either mass flow rate or detailed macroscopic spray parameters transitioning in and out of the flash-boiling regime from this widely used injector. These parameters are then compared with a commercially available injector at the same set of test points. It is found that the injector design has a significant effect, and the spray collapse (flare flash boiling) point depends strongly on the details of the injector-hole design. The tests detailed in this paper provide a comprehensive data set for future model validation on the ECN Spray M injector.

## Methodology

### Mass Flow Rate Measurement

The injection mass flow rate was measured using a pressure-based method immediately upstream of the injector and is based on the single-pressure-transducer method proposed by Ferrari et al.^27^ The method exploits the propagation of pressure waves in the liquid fuel line generated by rapid changes in the fluid velocity during injector opening and closing. When the injector needle opens, the sudden acceleration of the liquid produces a transient pressure disturbance that propagates through the fuel line at the local speed of sound. The magnitude of this pressure disturbance is directly related to the change in momentum of the liquid and, therefore, to the instantaneous mass flow rate through the injector.

The derivation assumes one-dimensional wave propagation in a slender fuel line and applies conservation of mass and momentum across the traveling pressure wave. Considering a control volume moving with the wavefront, a relationship can be obtained between the pressure rise generated by the injection event and the corresponding change in fluid velocity. Assuming the fuel line behaves as a rigid pipe and that elastic deformation of the wall is negligible, the resulting expression for instantaneous mass flow rate is

1 $$ṁ=-A\frac{\Delta p}{c}$$

where *ṁ* is the mass flow rate, *A* is the pipe cross-sectional area, *c* is the speed of sound of the fluid, and Δ*p* is the pressure change due to the injection event.

The method, therefore, converts the measured pressure-wave amplitude directly into the mass flow rate using the acoustic properties of the fuel and the geometry of the pipe. Physically, the pressure signal represents the momentum transfer associated with the injection event, while the speed of sound governs the propagation velocity of the compressive wave through the liquid column. Because the technique measures the transient hydraulic response of the fuel line, it is capable of resolving the full time-dependent injection rate profile, including injector opening dynamics and short-duration oscillatory behavior.

Accurate determination of the local speed of sound (*c*) is critical, because the calculated mass flow rate is inversely proportional to *c*. Unlike conventional hydrocarbon fuels, the speed of sound of liquid ammonia varies significantly with both temperature and pressure due to its relatively high compressibility and proximity to saturation conditions under the test conditions investigated. For this reason, the speed of sound was not treated as a constant value. Instead, thermodynamic property data for liquid ammonia (NIST Webbook^28^) were used to evaluate *c* at the measured fuel temperature and injection pressure immediately prior to each injection event. These data were cross-referenced against experimental data from Dubberke et al.^29^ The pressure dependence of c over the pressure fluctuations occurring during injection was comparatively small, and therefore, a single preinjection value of *c* needed to be used for each injection condition.

Injection pressure was monitored using a GEMS 3100 series transducer at low frequency. For mass flow rate measurement, high-frequency pressure (and hence Δ*p*) was measured using a single absolute pressure transducer (Kistler 4007D) with a range of 0–250 bar and a frequency response of >60 kHz through a Kistler 4624A amplifier. This high-speed sensor was mounted approximately 350 mm upstream of the injector, with a through-bored injector mount on a 4 mm inner diameter pipe, with a further 3 m of pipework upstream before the fuel pump, both designed to minimize pressure wave reflections, which interfere with the mass flow measurement technique.^27^ Data from the high-speed pressure transducer, alongside other parameters such as injection electrical actuation signals, were logged using a PicoScope 6000E series data acquisition system operating at a sampling frequency of 100 kHz.

### Injectors and Test Conditions

Table 1 shows the specifications of the two injectors tested in this work. The first injector is a Bosch six-hole injector (HDEV 5.1), which has been fully described in Mohd Murad et al.,^30^ and its performance using ammonia as a working fluid has been reported in Shen et al.^17^ The second injector was an eight-hole injector (ECN Spray M). The ECN Spray M has *C*_d_ values in the range 0.47–0.52 reported in the literature,^31,32^ *C*_d_ values are unavailable for the HDEV 5.1 injector. Both injectors have their origins as gasoline direct injection (GDI) injectors, although modifications, primarily to their seals, have been made to enable their use with ammonia. The injectors were actuated for 20 consecutive shots at a frequency of 1 Hz.

**Table 1:** Injector Specifications for the ECN Spray M and HDEV 5.1 Injectors

	ECN Spray M	HDEV 5.1
Manufacturer	BorgWarner	Bosch
Number of holes	8	6
Hole diameter (μm)	170	210
L/D (−)	1.4	1.4
Counterbore diameter (μm)	400	440
Counterbore length (μm)	450	330

The test conditions were designed to explore the range of spray morphologies expected from nonflash boiling to full-flare flash boiling. Results are reported using the (dimensionless) saturation-to-ambient pressure ratio, defined as

$$r_{P}=\frac{\mathrm{saturation vapour pressure}}{\mathrm{ambient pressure}}=\frac{P_{\mathrm{sat.}}}{P_{\mathrm{amb.}}}$$

Note that this work has adopted a new definition of *r*_*p*_ compared to previous publications from these authors, which was adopted in other works in the literature.^14^ However, the recent convention^33–35^ has been to use the saturation-to-ambient pressure ratio (sometimes referred to as the superheat degree) rather than the ambient-to-saturation pressure ratio (i.e., the inverse), and this definition has been adopted here—not least because it highlights the flare flash boiling area with high numerical values, which is a substantial area of investigation in this work.

The ambient pressures, therefore, varied from 1–15 bar, with increments as low as 0.5 bar in transition regions between flash boiling and nonflash boiling sprays (i.e., at values of *r*_*p*_ ≥ 1). Anhydrous liquid ammonia was pressurized to two injection pressures (100 and 150 bar), and all data were collected using the same injection duration (2 ms) from both injectors. The ambient gas pressures used are not identical for the two injectors and the precise points tested are shown in Figure 1. For all test conditions, the ammonia (fuel) temperature and nitrogen (chamber) temperature were ambient; measured as 17 ± 1^◦^C.

**Figure 1:**
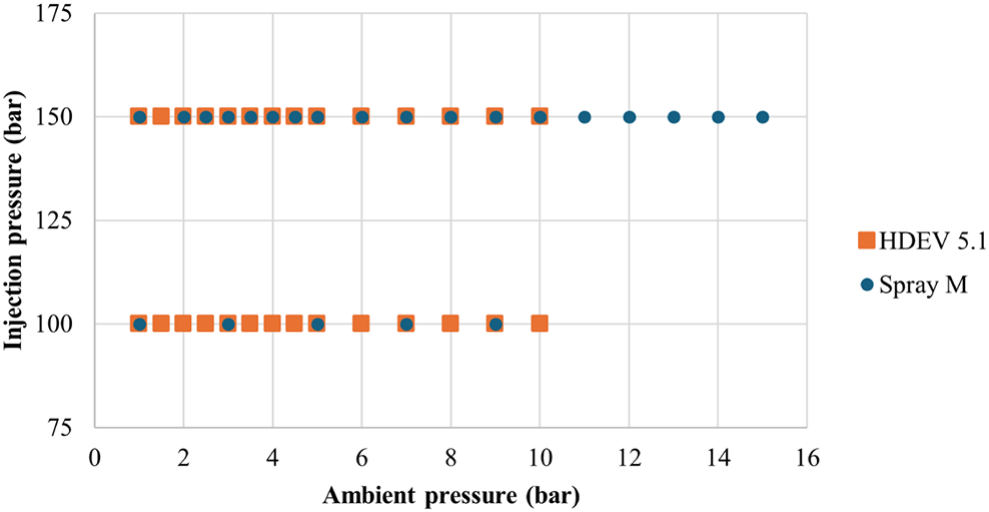
Test conditions for both HDEV 5.1 and Spray M injectors. All conditions had both spray image and mass flow data taken.

### Spray and Image Analysis

Ammonia sprays from the two different injectors were measured in a constant-volume chamber. This chamber was filled to various ambient pressures using nitrogen as a non-reactive ambient gas. This chamber, setup, and the image processing methodology have been comprehensively described in previous work,^11,17^ so only a brief summary is given here for convenience. The chamber is cylindrical in shape and has three axes of optical access, although in this work, only one axis is used. This axis has two fused silica windows that have an 80 mm visible diameter. A schematic of the rig, from the camera view, including details of the pressure measurement locations used for the mass flow rate analysis, is shown in Figure 2.

**Figure 2:**
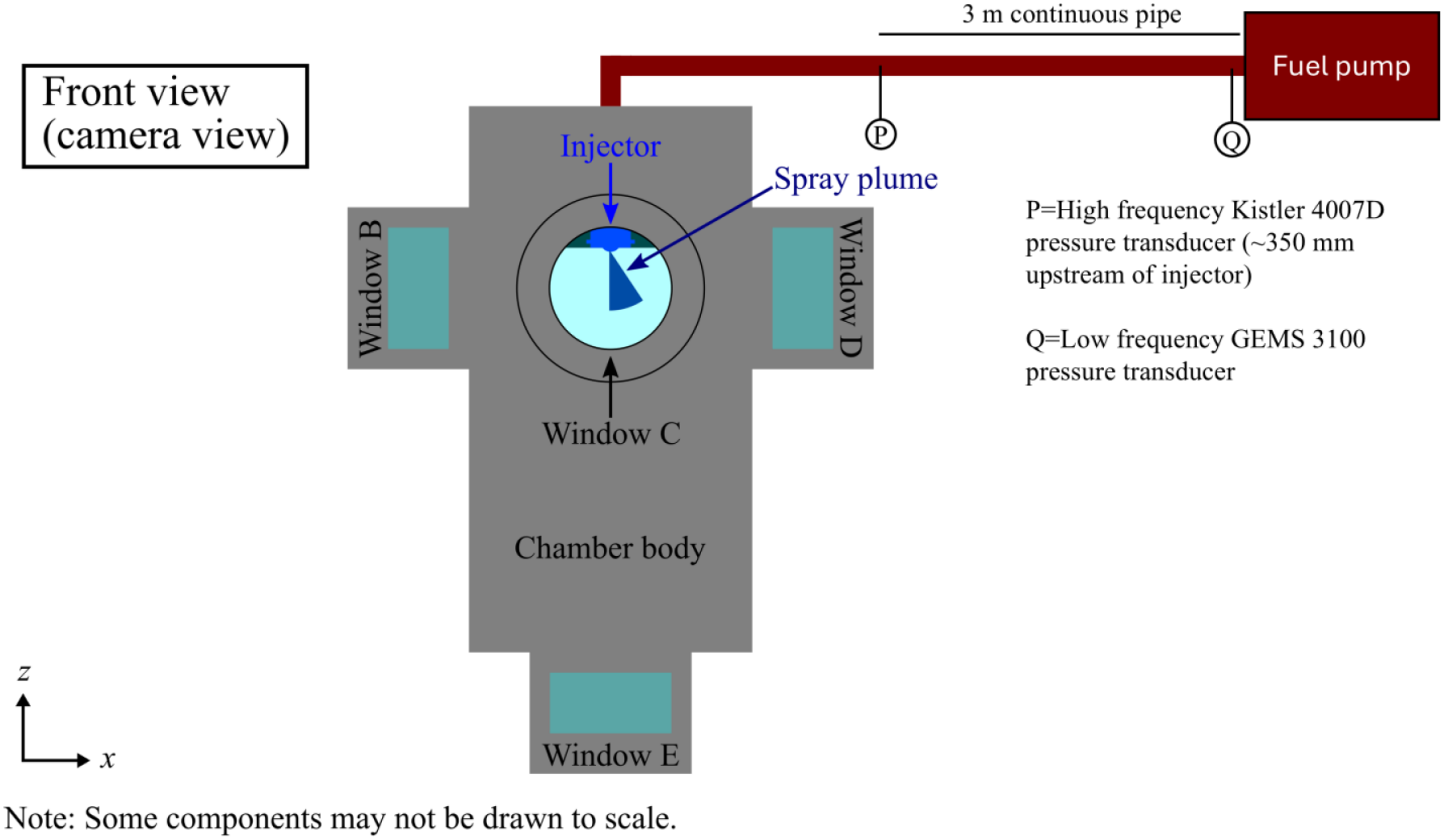
A schematic of the test rig showing the general arrangement and details of where the pressure measurements used for mass flow rate analysis were taken. Note: the schematic is not to scale.

The sprays were back-illuminated with a white LED through a diffuser screen, which gives a constant background illumination to the images. The images are hence effectively shadowgraphs. These were captured by a Photron FASTCAM-1024PCI 100 K high-speed camera, which was triggered to record 10 ms before injection giving ten blank images prior to injection. The camera recorded images at a rate of 10,000 frames per second (FPS), at an exposure of 10 μ*s* and a resolution of 256 × 272 pixels. This gives a resolution of 0.25 mm per pixel.

Spray macroscopic parameters, spray tip penetration length, tip penetration speed, and spray cone angle, were computed from the backlit images using an algorithm based on the SAE J2715 standard.^36^ To isolate spray features from the background of the images, the 10 blank images that are recorded immediately before the start of the energizing current are subtracted from each subsequent image, so that the resulting background-subtracted image contains only spray features. The spray penetration length is then computed by taking the furthest point of that background-subtracted image compared to the spray tip (which serves as the origin for these calculations). The tip penetration speed is calculated by comparing the tip penetration lengths between spray images (0.1 ms intervals), hence, tip penetration speed information is only available after two consecutive tip penetration lengths have been calculated. Spray cone angle is calculated by drawing two circles centered on the injector tip (origin) at radii of 5 mm and 15 mm. Points are then drawn where these two circles intersect the spray, and lines are drawn between these two points on their respective spray edges. The spray cone angle is the angle between the two lines. Hence, spray cone angle data are only available once the spray has penetrated at least 15 mm.

## Results and Discussion

### Mass Flow Rate Measurements

Figure 3 (lower) shows the mass flow rate and cumulative mass of single-liquid ammonia injection events. This example test condition has a chamber pressure of 1 bar, a nominal injection pressure of 150 bar, and an injection duration of 2 ms. Under these conditions, the mean injected mass is 26.63 mg and the 95% confidence interval over the 20 injections is 0.67 mg. This error combines both the uncertainty in the method and the injection-to-injection variation. Individual injections are also shown, with the faint lines in Figure 3 showing each individual injection (20 in total) and the bold line showing the mean. The upper part of Figure 3 shows the pressure signal (similarly bold for the mean and faint lines for each individual injection) in red that is used to obtain the mass flow rate, and the injector energizing current in gray.

**Figure 3:**
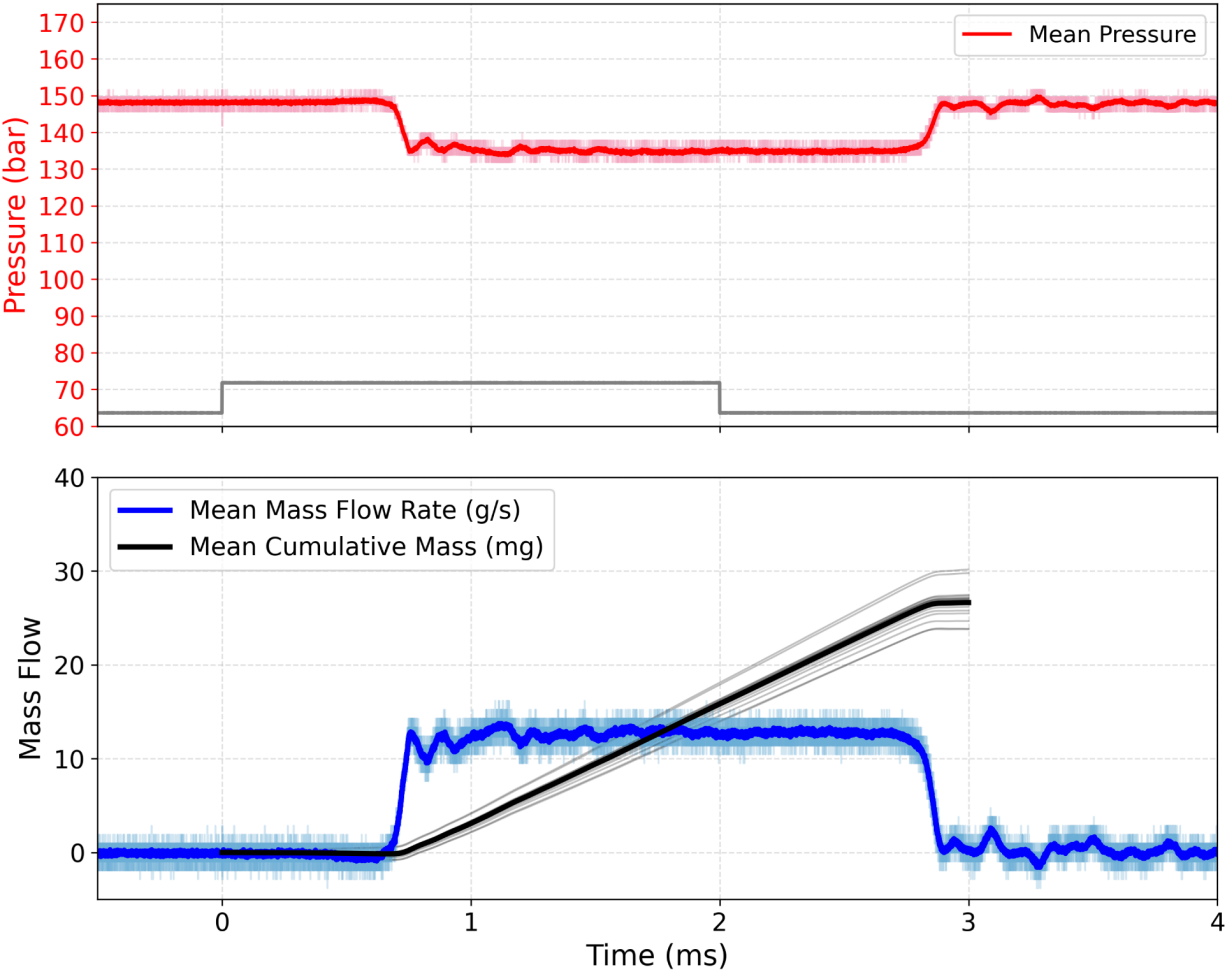
Example mass flow rate and cumulative mass of ammonia injected from the ECN Spray M injector at a nominal injection pressure of 150 bar and an injection duration of 2 ms (lower plot). The pressure signal (red) that is used to obtain the mass flow rate, and injector energizing current (gray) are shown in the upper part of the plot. The bold lines show the mean, and the fainter individual lines show each of 20 repeats at this test condition.

Figure 3 and Table 2 illustrate characteristic patterns for all of the ammonia injections in this work. There is an approximately 0.7 ms delay between the energizing signal and the start of the injection event. Part of this can be attributed to the distance between the pressure transducer making the measurements and the injector, however, this would only account for approximately a 0.25 ms delay. The remainder (≈0.45 ms, depending on the injector) can be attributed to hydraulic delay, which is caused by the ammonia viscosity, the needle lift, and the nozzle type.^37^ This is confirmed by analyzing the spray images, where visible injection is seen between 0.4 and 0.5 ms after the injection signal; this is the maximum resolution of the spray images.

**Table 2:** Delays between Start of Energising Signal and the Measured Start of Injection

Injection Pressure	ECN Spray M	HDEV 5.1
100 bar	688 ± 4 *μs*	718 ± 7 *μs*
150 bar	675 ± 7 *μs*	716 ± 3 *μs*

It is also observed that the injection takes the characteristic “square wave” or “top hat” shape common to this type of application.^38^ There are also some oscillations observed at the start of the hydraulic injection (from 0.75 to 1.2 ms after the energizing current starts), which are likely associated with needle oscillations as it reaches its peak lift point.^39^ The oscillations in the signal after the end of injection (after 2.8 ms) are associated with reflecting pressure waves and do not represent real flow out of the injector, which is closed from this point. Rate shape data from every test point is available in the data associated with this paper.

Figure 4 shows the total mass injected per injection plotted against the net injection pressure for all test points with 150 bar target injection pressure (left-hand plot) and a comparison of mass flow rates of injection from a flash boiling and a nonflash boiling case; all with the ECN Spray M injector. The error bars show the 95% confidence interval over the 20 injections at each test point. The 95% confidence intervals, combined uncertainty of the method, and the injection-to-injection variations are low, with an average 95% confidence interval of 2% of the measurement value (and a maximum of 3.1% of the measurement value). There is no significant difference between the lowest and highest net injection pressure (i.e., full flash boiling and nonflash boiling cases). This is in contrast with some data in the literature, which observe a reduction in mass flow rate with flash boiling,^40^ but in line with other studies that do not observe such a difference.^41^ It is clear, when considering the rate of injection profiles (right-hand plot in Figure 4) that this is because there is no significant difference between these conditions throughout injection rather than any netting-off effects through the injection period. Similar trends are observed at a 100-bar injection pressure (not shown for brevity).

**Figure 4:**
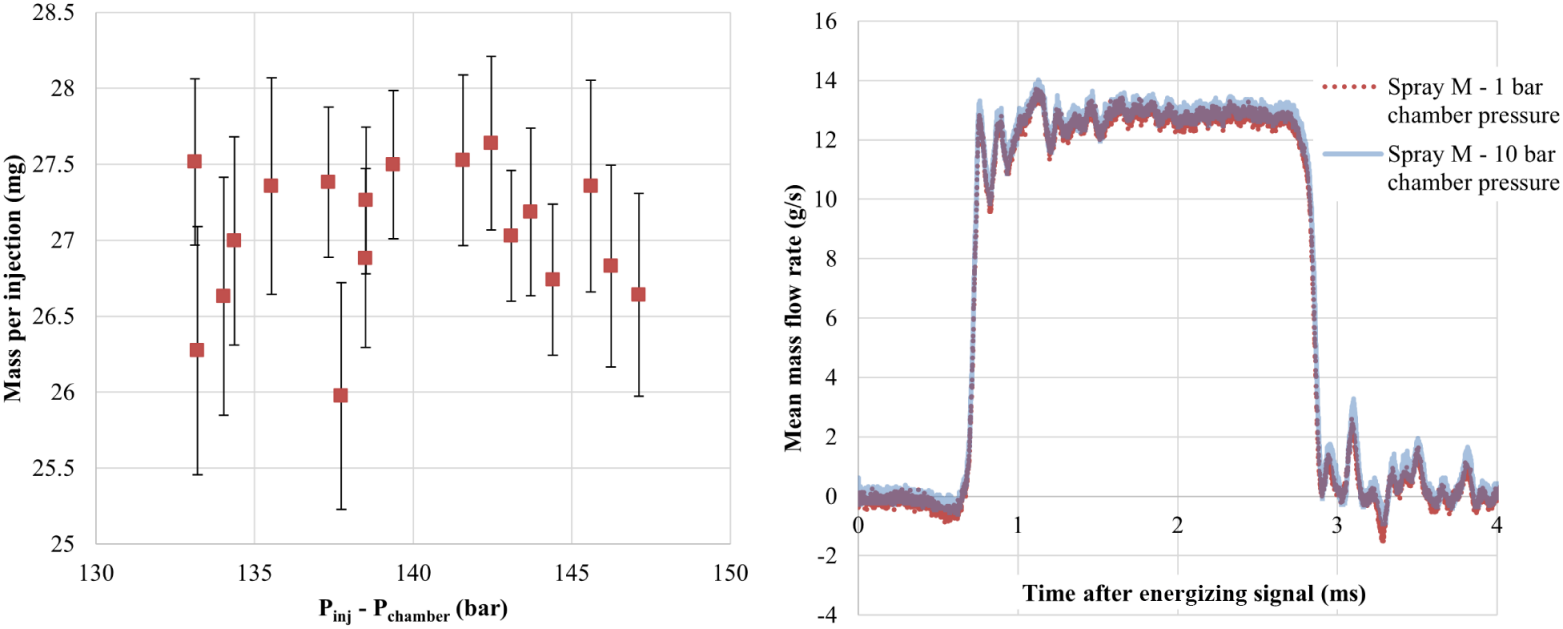
Mass of ammonia injected per injection with the ECN Spray M injector for all points with 150 bar target injection pressure (L). The error bars represent ± 95% confidence interval. Comparison between mean mass flow rates of injection from the Spray M and HDEV 5.1 injectors at a nominal injection pressure of 150 bar and injection duration of 2 ms for a flash boiling (1 bar chamber pressure) and nonflash boiling (10 bar chamber pressure) cases (R). The plot shows the mean of 20 repeats at this test condition.

#### Injector Comparison

The mass injected per injection from each of the injectors at both injection pressures is shown in Figure 5 at an example condition with a 5-bar ambient gas pressure. With the ECN Spray M injector, a 23% reduction in injected mass is observed with injection pressure reducing from 150 to 100 bar. This is very close to the reduction observed with injection pressure reducing from 150 to 100 bar with the HDEV injector of 21%. Overall, the HDEV injector injects less mass (around 10% at 150 bar and 13% at 100 bar) at a given test point, which given its 6-hole design (compared to 8-holes for ECN Spray M) is perhaps not surprising.

**Figure 5:**
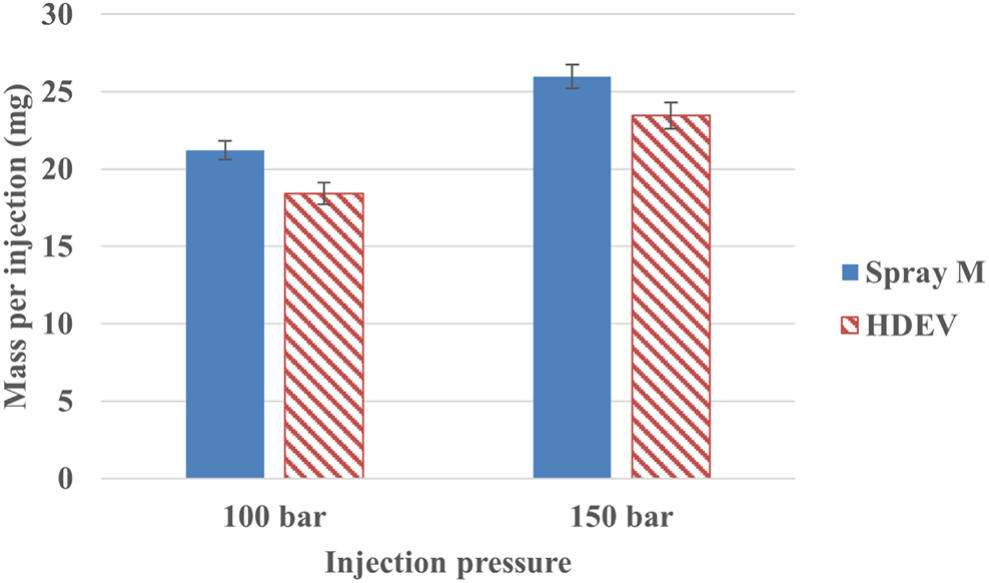
Mass of ammonia injected per injection at 100 and 150 bar target injection pressures, 5 bar chamber pressure for ECN Spray M and HDEV 5.1 injectors. The error bars represent ±95% confidence interval.

The mean mass flow rates from 20 injections, each from the Spray M and HDEV injectors at 150 bar injection pressure, are shown in Figure 6; many similarities are observed. One key difference, however, is that with the HDEV injector, a small amount of negative mass flow is observed at the start of injection; this is associated with the needle lift, which is not observed with the ECN Spray M injector. In addition, it is now clear that the lower total injected mass from the HDEV injector observed in Figure 5 can be attributed to two features. First, there is a lower peak flow rate from the HDEV injector compared to the Spray M injector, consistent with it having two fewer nozzles. Second, the total injection duration for the HDEV injector is also, on average, slightly shorter: a 3.9% reduction (2.14 ms for HDEV and 2.23 ms for Spray M).

**Figure 6:**
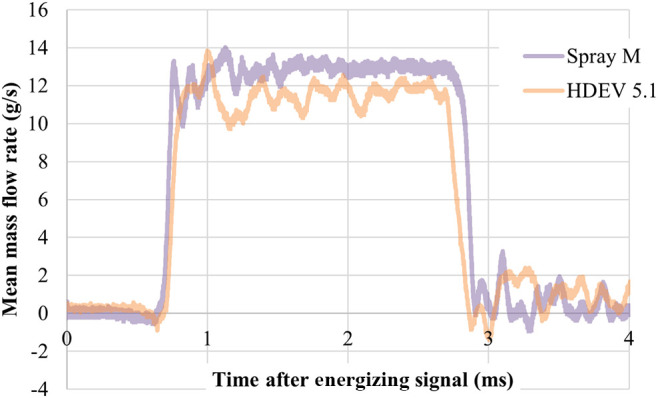
Mean mass flow rates from the Spray M and HDEV 5.1 injectors at a nominal injection pressure of 150 bar and an injection duration of 2 ms. The plot shows the mean of 20 repeats at this test condition.

### Qualitative Analysis of Spray Images

Snapshot spray images from both injectors (HDEV 5.1 top row, ECN Spray M bottom row) at different ambient pressures are shown in Figure 7 at 1.25 ms aSOI (approximately halfway through the hydraulic injection). Throughout this section, the results are displayed as time after start of injection (aSOI), which is determined by the first visible spray droplets leaving the injector and is ≈0.45 ms after the start of the electrical injection signal. The effect of the HDEV 5.1 injector’s asymmetrical hole pattern, where two of its nozzles point more radially, compared to the other four, which point more axially downward, is clear. A visible separation of the spray plumes at higher ambient pressures, when the spray was in nonflash boiling region is clearly seen. Plume merging (spray collapse) appears only at extremely low ambient pressures (1 bar), when the spray clearly undergoes flare flash boiling.

**Figure 7:**
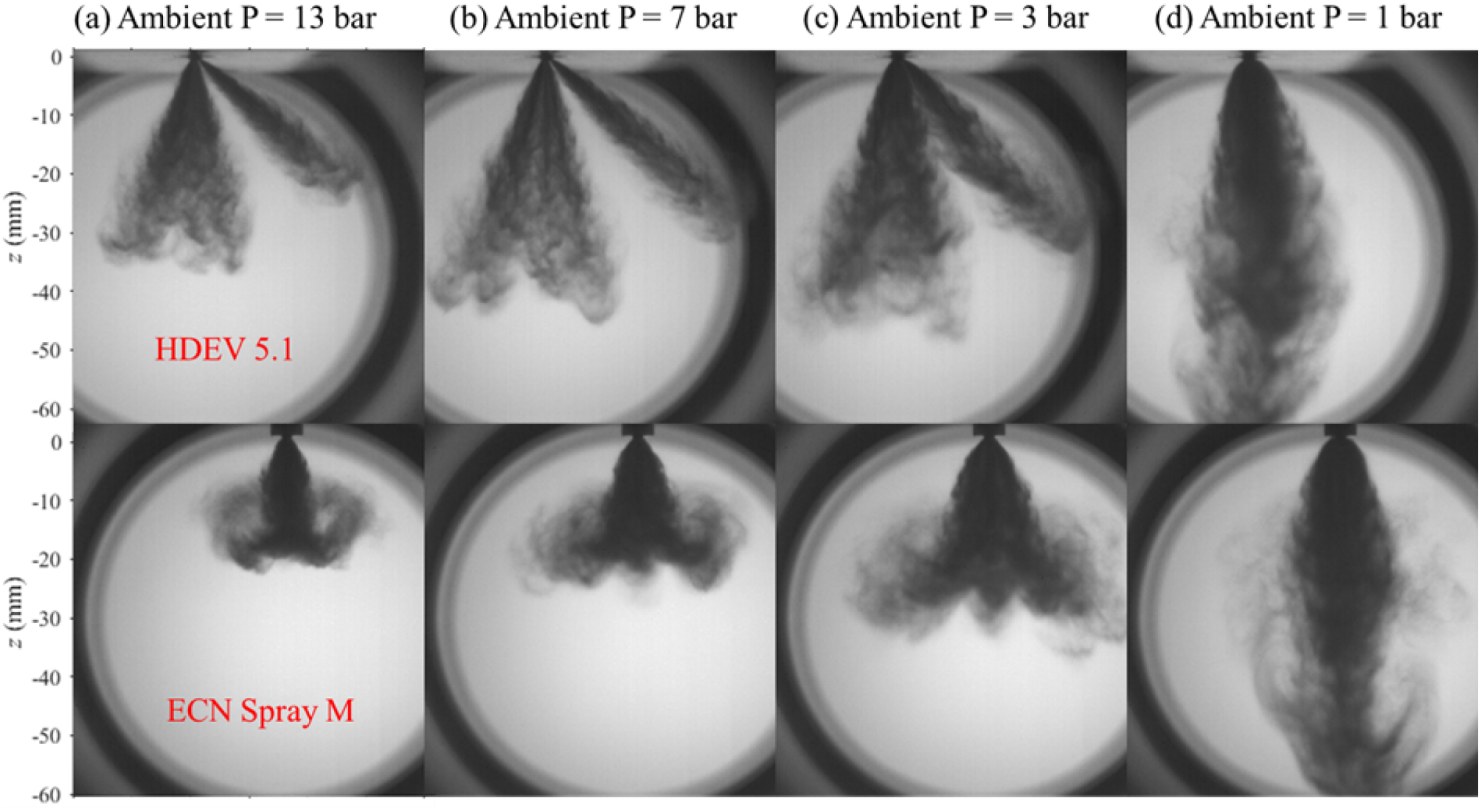
Ammonia spray images recorded at 1.25 ms aSOI under (a) 13 bar, (b) 7 bar, (c) 3 bar, and (d) 1 bar ambient pressures. The images on the top row are from the HDEV 5.1 injector, and the bottom row are from the ECN Spray M injector. The saturated vapor pressure at the tested ambient temperature is 8.5 bar. The injection pressure for these test conditions was 150 bar, and duration was 2 ms.

On the other hand, the spray morphology of the ECN Spray M injector looks very different to the HDEV 5.1 injector; the plumes from each nozzle were much closer to each other, and even at high ambient pressures, there is no clear plume separation—the spray almost collapsed immediately after leaving the nozzles. This is likely due to the axi-symmetric hole pattern from this injector and the increased number of holes (8 for ECN Spray M vs 6 for HDEV 5.1). The flash boiling mechanism at extremely low ambient pressures (1 bar), nevertheless, still substantially alters the morphological behavior of the collapsed spray. In these cases, the spray morphologies between the ECN Spray M and HDEV 5.1 injectors look similar. It can also be seen that, except at 1 bar ambient pressure (the most aggressive flash boiling), the penetration length from the ECN Spray M injector is much shorter than the HDEV injector, and the recirculation near the plume tip is much stronger. Considering that the injection and the ambient pressures were kept the same, the effect of ambient drag will be similar. The differences will arise due to the differing hole sizes (although this difference is small) between the injectors, changing the spray momentum. A bigger effect is expected to arise due to the effect of in-nozzle cavitation, which will likely be stronger from the ECN Spray M injector, due to its longer counterbore design, and this prevents the liquid part of the spray from traveling further and achieving greater ambient gas entrainment.

### Quantitative Analysis—Spray M Injector

By analyzing the spray images, quantitative parameters such as spray penetration length, penetration speed, and cone angle are obtained. Figure 8 shows the spray penetration length, spray penetration speed, and spray cone angle for the ECN Spray M injector over time for all test points for this injector. The left-hand column of Figure 8 is at an injection pressure of 100 bar, and the right-hand column at 150 bar injection pressure. Each marker labels the mean value of the results from 20 consecutive shots in a single test run, and the error bars represent one standard deviation (σ).

**Figure 8:**
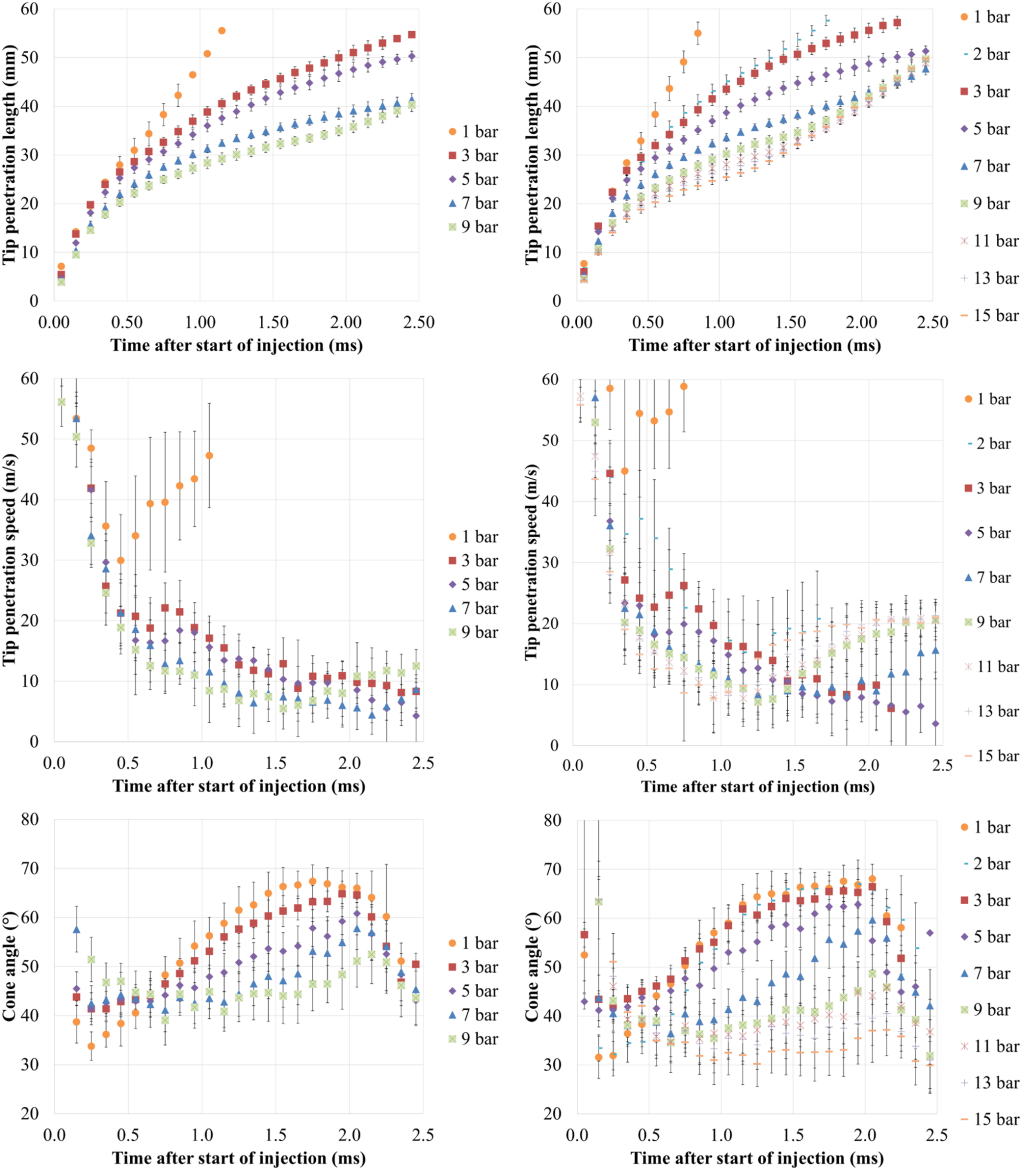
Macroscopic spray parameters of the ECN Spray M injector as functions of time at 100 bar (left-hand column) and 150 bar (right-hand column) injection pressure, injection duration 2 ms. Tip penetration length (top), tip penetration speed (middle), and spray cone angle (bottom). Error bars correspond to ± σ.

For the spray tip penetration length, 60 mm represents the viewable limit of the window, so data beyond this point cannot be observed. As would be expected, the penetration length decreases monotonically with increasing ambient pressure due to the increased drag from the higher-density ambient gas. The spray collapse observed in Figure 7 leads to a substantially greater penetration length at 1 bar ambient pressure (blue diamonds) compared to any other ambient pressure, even 2 bar. Comparing the left-hand and right-hand spray penetration length figures in Figure 7, it is clear that the higher injection pressure (150 bar) leads to a higher penetration length sooner, with the 1 bar ambient pressure case taking approximately 0.4 ms longer to reach a penetration length of 50 mm and higher ambient pressure cases all being slower to reach a given penetration length and reaching lower final penetration lengths.

For spray tip penetration speed, lower speeds are observed at all test conditions at 100 bar injection pressure (left-hand column in Figure 8) compared to 150 bar injection pressure (right-hand column in Figure 8). This is consistent with the penetration length results. When the impact of ambient pressure on the results is considered, there are three groups of data. The first, at the lowest ambient pressure (1 bar), where flare flash boiling is observed, shows extreme, rapid reacceleration at around 0.5 ms aSOI (middle parts of Figure 8). There is also some more limited reacceleration visible in the 3 and 5 bar ambient pressure cases in Figure 8, although not at as high a magnitude as the 1 bar case. This is perhaps indicative that, under these low ambient pressures, there is not enough energy available from the ambient gas to evaporate the ammonia. The midpressure cases (2.5–7 bar ambient pressure) do not experience any reacceleration, and the penetration speed continues to fall monotonically. The highest ambient pressure cases (8–15 bar) exhibit a later reacceleration at around 1.3–1.4 ms aSOI.

For spray cone angle, once the initial cone angle has stabilized (at around 0.3 ms aSOI) at high ambient pressures (>11 bar) the cone angle does not vary much, however, as the flash boiling regime is encountered, the cone angle increases after initial stabilization, and in the lowest ambient pressure cases, where full plume collapse is observed, the cone angle increases by around 20^◦^ compared to the initial cone angle at 0.3 ms aSOI. In addition, overall, flash boiling increases the cone angle by around 30^◦^. Cone angle is broadly unaffected by injection pressure.

### Quantitative Analysis in the Pressure Ratio Domain

Previous work^17^ has shown that the saturation-to-ambient pressure ratio (*r*_p_) has a significant effect on the morphology of ammonia sprays. For this reason, the data in Figure 8 have been replotted in terms of (*r*_p_) so that the effect can be clearly observed. This is shown in Figure 9, and note that, on these plots, error bars are omitted for clarity. These data are shown solely at 150 bar injection pressure for brevity, however, the results at 100 bar injection pressure show no material difference. Any point with an *r*_p_ > 1 will theoretically undergo flash boiling as the ambient pressure is below the saturation pressure. However, for the ECN Spray M injector, different behavior is observed. Spray penetration length and speed are highest when *r*_p_ = 2.43, and the increase in spray cone angle (an indication of flash boiling) plateaus above this *r*_p_. Previous work^17^ has indicated that ambient drag dominates the spray behavior below this value of *r*_p_, and that is what is again observed here with the ECN Spray M injector (green-colored areas on Figure 9). With *r*_p_ > 2.43 (blue-colored areas on Figure 9), evaporation (thermal breakup) begins to dominate the spray behavior leading to a plateauing in penetration length, speed, and cone angle. This regime continues until *r*_p_ = 3.40. For *r*_p_ > 3.40 (red-colored areas on Figure 9), as seen in Figure 7(d), the spray collapses into a single plume, and undergoes flare flash boiling, (which leads to smaller spray particle sizes^11,42,43^ and this dramatic change leads to different aerodynamic properties; the penetration length and speed both reduce, associated with reduced spray momentum from smaller droplet sizes, and the spray cone angle undergoes a reduction, similar to the cone-collapse morphology observed with this injector in Qenawy et al.^22^

**Figure 9:**
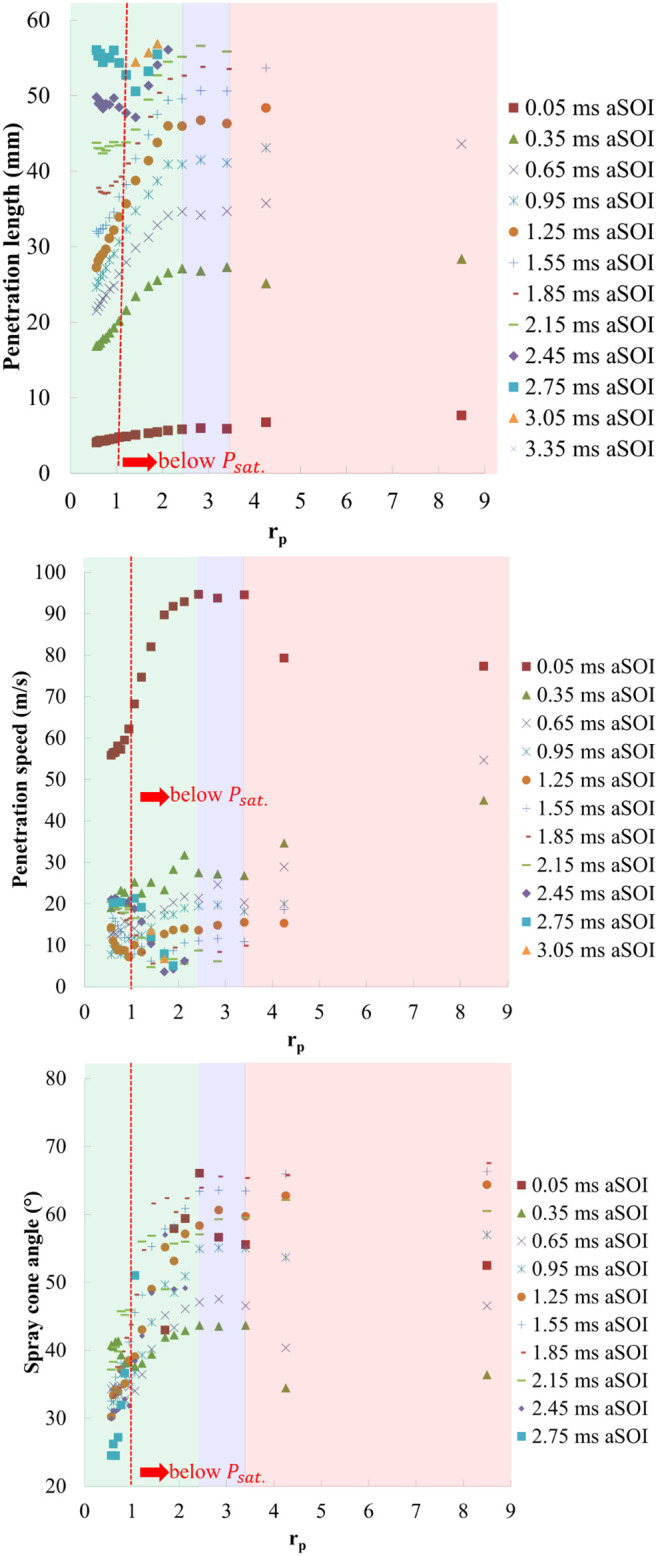
Tip penetration length (upper), tip penetration speed (middle), and spray cone angle (lower) at various times after the start of injection (aSOI) as a function of saturation-to-ambient pressure ratio (*r_p_*) at 150 bar injection pressure.

#### Injector Comparison

Figure 10 shows the comparison in tip penetration length (top), tip penetration speed (middle), and spray cone angle (bottom) between the ECN Spray M injector and the HDEV injector. Throughout the injection event, it can clearly be seen that the HDEV injector has a higher tip penetration length and much higher initial tip penetration speed, depending on the ambient pressure, up to 25 mm further penetration at a given time. This suggests that there is more momentum in the spray from the HDEV injector, which may lead to better (further) spray distribution but indicates that less evaporation is occurring, (although this is conjecture), suggesting the presence of larger spray droplets from the HDEV injector. With regard to cone angle, shown in Figure 10 (lower), there is much more variation with ambient pressure seen with the ECN Spray M injector compared to the HDEV injector, again indicating that evaporation and breakup have a bigger role with this injector.

**Figure 10:**
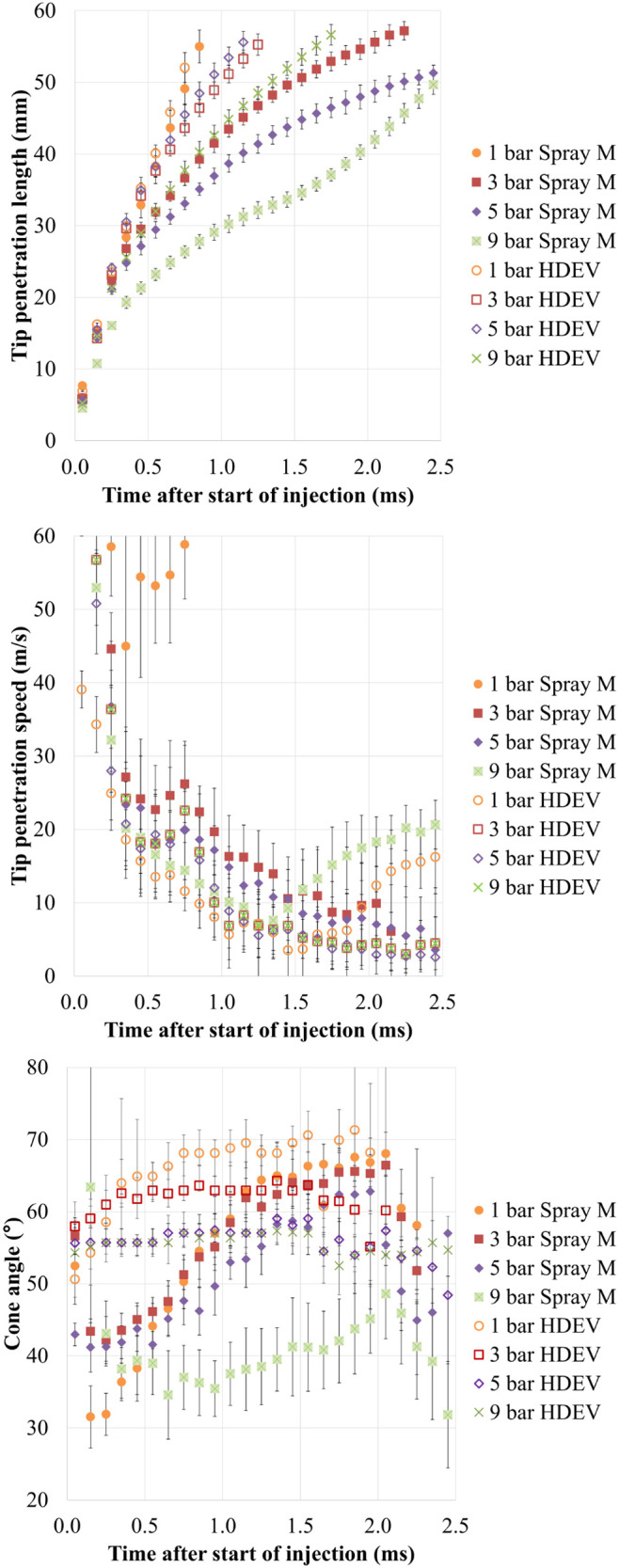
Comparison of tip penetration length (upper), tip penetration speed (middle), and spray cone angle (lower) between ECN Spray M injector and HDEV 5.1 injector at 150 bar injection pressure, 2 ms injection duration.

The data for the HDEV injector in the *r*_p_ space have already been presented in previous work,^17^ however, it is of interest to compare where the transition pressure ratios are between the three regions (drag dominant, evaporation dominant, and flare flash boiling). These are listed in Table 3. The ECN Spray M injector design results in flare flash boiling occurring at a lower *r*_p_, most probably due to its longer counterbore design promoting cavitation. However, at higher ambient pressures, the drag region dominates for longer—the transition region being entered at a higher *r*_p_ with the ECN Spray M injector compared to the HDEV injector.

**Table 3:** Ranges of the Three Regions of Spray Morphological Behaviour and Evaporation Mechanisms for Both Injectors at 150 Bar Injection Pressures

Region	Evaporation mechanism	ECN Spray M	HDEV
Drag dominant	External heat transfer	*r*_*p*_ ≤ 2.43 (*P*_amb._ ≥ 3.5 bar)	*r*_*p*_ ≤ 1.89 (*P*_amb._ ≥ 4.5 bar)
Evaporation dominant or mild flash boiling	Both	2.43 < *r*_*p*_ < 3.40 (2.5 < *P*_amb._ < 3.5 bar)	1.89 < *r*_*p*_ < 4.35 (2.0 < *P*_amb._ < 4.5 bar)
Spray collapse or flare flash boiling	Superheated evaporation	*r*_*p*_ ≥ 3.40 (*P*_amb._ ≤ 2.5 bar)	*r*_*p*_ ≥ 4.35 (*P*_amb._ ≤ 2.0 bar)

## Discussion

It is interesting to compare the results from Figure 7 to those of other publications in the literature using the same injectors but using different working fluids. Mohd Murad et al.^30^ have previously published with the HDEV 5.1 injector using a mixture of 40% *n*-heptane and 60% iso-octane as the working fluid. Similarly, the ECN Spray G injector (virtually identical to the ECN Spray M injector) is well-characterized in the literature, but the results and images from Hwang et al., using 100% iso-octane as the working fluid, are a particularly useful comparison.^44^ The comparison is listed in Figure 11. Simply comparing chamber pressure conditions in the literature, the behaviors appear very different, which would be expected given the substantially different fluid properties. However, if comparing conditions at the same saturation-to-ambient pressure ratios (*r*_p_), as shown in Figure 11, the qualitative comparison can be very close; the spray morphologies for HDEV 5.1 look very similar and those for ECN Spray M do at moderate flashing. This suggests that saturation-to-ambient pressure ratio (*r*_p_) continues to be a very useful metric for comparisons across different working fluids. However, differences arise when comparing reductions in penetration length under flashing with HDEV 5.1 and spray morphology with ECN Spray M. These are likely due to the high enthalpy of vaporization of ammonia compared to the fuels used in Mohd Murad et al. and Hwang et al.This is a parameter that is not captured by *r*_p_, and so retaining a wholistic approach to sprays from ammonia, rather than extrapolating from other fuels, remains important.^30,44^ ^,^

**Figure 11:**
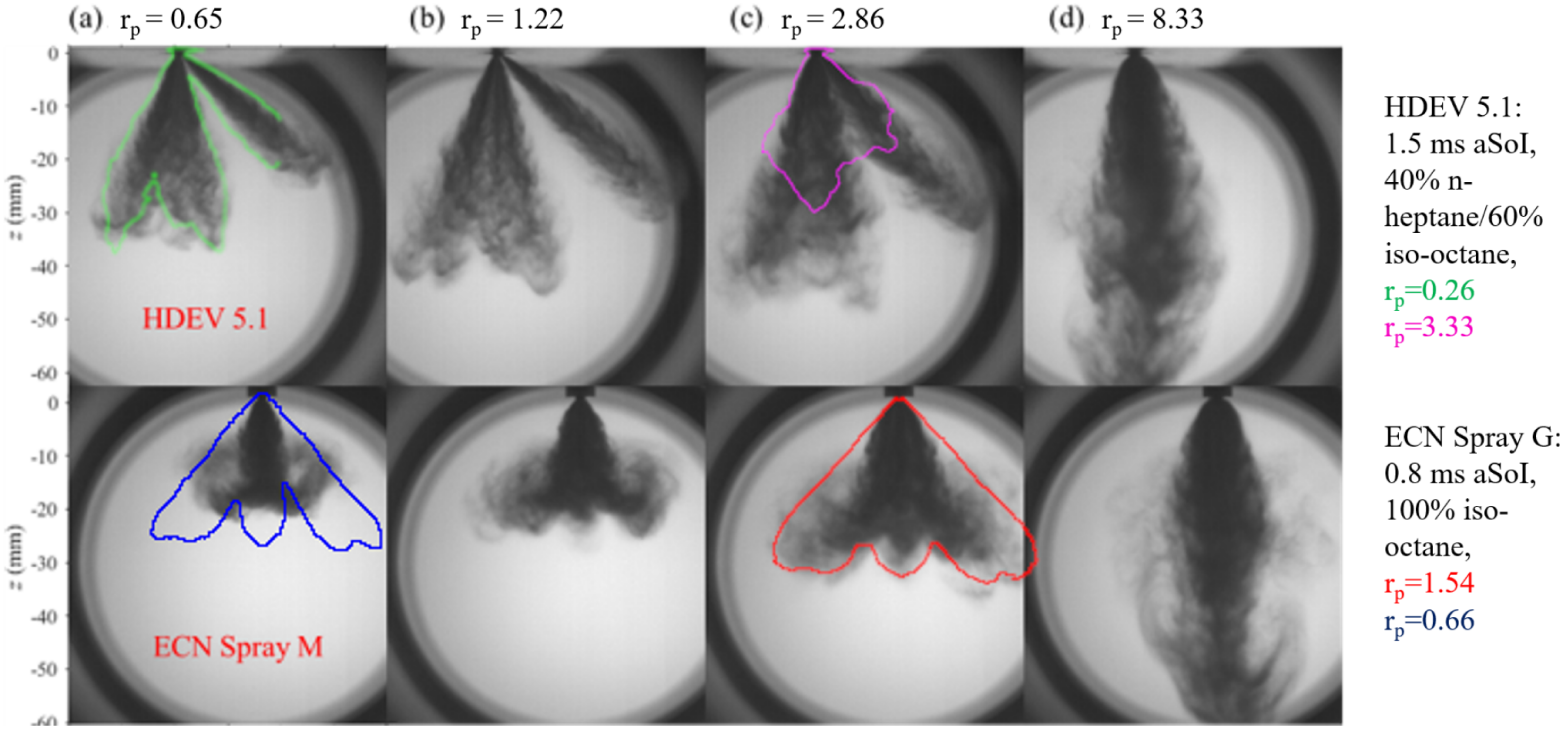
Figure 7 is overlaid with literature data from Mohd Murad et al.^30^ (top row) and Hwang et al.^44^ (bottom row) at the closest available conditions published in those works; conditions shown on the right of the figure. As a reminder, ammonia spray images at 1.25 ms aSOI.

Recent studies on ammonia spray behavior using ECN Spray M multihole injectors have reported trends that both support and contrast with the findings of the present work. Qenawy et al.^22^ investigated near-critical and supercritical ammonia injections using the ECN Spray M injector and observed that increasing ambient pressure progressively altered the spray morphology from bell collapse to cone collapse and finally to distinct individual jet structures. In contrast, the present study demonstrates that the ECN Spray M injector exhibits flare flash boiling and plume collapse primarily at high saturation-to-ambient pressure ratios, with clear transitions between drag-dominated, evaporation-dominated, and flare flash boiling regimes. Qenawy et al. attributed the suppression of collapse at elevated ambient pressure to enhanced aerodynamic resistance and reduced near-nozzle jet expansion, which aligns with the findings in this work.

Similarly, Sonawane et al.^45^ used the ECN Spray G injector (very similar to the Spray M injector) to investigate optical depth and multiple scattering in superheated ammonia sprays. Their results showed that increasing flash-boiling intensity promoted plume interaction, spray collapse, and dense near-nozzle regions with high optical depth. Although these observations are qualitatively consistent with the flare flash-boiling regime identified in the present study, important differences remain. Sonawane et al. concluded that sprays at a constant superheat degree (*r*_p_) could still evolve differently depending on ambient pressure and fuel temperature. The current work extends this concept by showing that injector geometry also strongly modifies the *r*_p_ transition boundaries themselves, with the Spray M injector entering flare flash boiling at lower *r*_p_ values than the commercial HDEV injector. Collectively, these comparisons highlight that ammonia spray behavior is governed not only by thermodynamic conditions but also by injector-specific geometric effects that strongly influence cavitation, breakup, and collapse dynamics.

## Conclusions

In this work, liquid anhydrous ammonia was injected from two multihole injectors into a nitrogen-filled constant volume chamber. Two injection pressures (100 and 150 bar) and a range of ambient gas conditions (1–15 bar) were tested. These ammonia sprays were imaged via shadowgraphy at 10,000 FPS, and the instantaneous mass flow rate was calculated via a pressure-based method. In addition to the qualitative comparisons of the spray images from the two injectors, macroscopic spray parameters: tip penetration length, tip penetration speed, and spray cone angle (in accordance with the SAE J2715 standard) calculated to enable quantitative comparisons. Mass flow rate and total injected mass were reported for all conditions. The results show that

- Qualitative comparisons of spray images reveal substantially different morphologies between the ECN Spray M injector and the HDEV 5.1 injector. These are to be expected, given the different injector designs and hole numbers. However, at the lowest ambient pressures, where the sprays undergo flare flash boiling, the spray morphologies look much more similar.
- For the first time, a detailed data set of tip penetration length, tip penetration speed, and spray cone angle is reported for the multihole ECN Spray M injector at a high resolution of *r*_p_ values across the flash boiling range.
- As previously reported, the sprays can be divided into three regions depending on the saturation-to-ambient pressure ratio (*r*_p_). These are a drag-dominant region at the lowest *r*_p_ values, an evaporation-dominant region in the middle of the *r*_p_ range, and flare flash boiling at the highest *r*_p_ values. However, flare flash boiling only occurs at much higher *r*_p_ values than the often assumed value of unity.
- While the presence of these three regions was consistent between the injectors, the *r*_*p*_ values at which the transition between them occurred was different—likely because of the presence of the longer counterbore in the ECN Spray M injector.
- For the first time, mass flow rate results from the ECN Spray M injector have been reported in the open literature, providing a powerful data set for modelers.

The results indicate that nozzle (injector) design is particularly important when using ammonia as the working fluid. Fortunately, by matching the saturation-to-ambient pressure ratio (*r*_p_), spray morphologies from ammonia sprays are qualitatively similar to those from traditional hydrocarbon sprays when using the same injector at the same conditions.

These results give further insight into injector design for ammonia sprays and provide useful data for computational model validation, enabling the design of ammonia injectors and combustion systems in the virtual world.

## Data Availability

All of the mass flow rate data and analyzed spray image data from this paper, including test conditions not presented in the figures for brevity, is available through the Oxford Research Archive (ORA).

## Abbreviations

aSOI: after Start Of Injection
CFD: Computational Fluid Dynamics
CO_2_: Carbon dioxide
ECN: Engine Combustion Network
FPS: Frames Per Second
GDI: Gasoline Direct Injection
LED: Light Emitting Diode
*r*_*p*_: Saturation-to-ambient pressure ratio (superheat degree)
SOI: Start Of Injection
σ: Standard deviation
